# The baseline pressure of intracranial pressure (ICP) sensors can be altered by electrostatic discharges

**DOI:** 10.1186/1475-925X-10-75

**Published:** 2011-08-22

**Authors:** Per K Eide, André Bakken

**Affiliations:** 1 Department of Neurosurgery, Oslo University Hospital, Rikshospitalet, Oslo, Norway; 2 Faculty of Medicine, University of Oslo, Oslo, Norway; 3 Department of Clinical and Biomedical Engineering, Oslo University Hospital, Rikshospitalet, Oslo, Norway

## Abstract

**Background:**

The monitoring of intracranial pressure (ICP) has a crucial role in the surveillance of patients with brain injury. During long-term monitoring of ICP, we have seen spontaneous shifts in baseline pressure (ICP sensor zero point), which are of technical and not physiological origin. The aim of the present study was to explore whether or not baseline pressures of ICP sensors can be affected by electrostatics discharges (ESD's), when ESD's are delivered at clinically relevant magnitudes.

**Methods:**

We performed bench-testing of a set of commercial ICP sensors. In our experimental setup, the ICP sensor was placed in a container with 0.9% NaCl solution. A test person was charged 0.5 - 10 kV, and then delivered ESD's to the sensor by touching a metal rod that was located in the container. The continuous pressure signals were recorded continuously before/after the ESD's, and the pressure readings were stored digitally using a computerized system

**Results:**

A total of 57 sensors were tested, including 25 Codman ICP sensors and 32 Raumedic sensors. When charging the test person in the range 0.5-10 kV, typically ESD's in the range 0.5 - 5 kV peak pulse were delivered to the ICP sensor. Alterations in baseline pressure ≥ 2 mmHg was seen in 24 of 25 (96%) Codman sensors and in 17 of 32 (53%) Raumedic sensors. Lasting changes in baseline pressure > 10 mmHg that in the clinical setting would affect patient management, were seen frequently for both sensor types. The changes in baseline pressure were either characterized by sudden shifts or gradual drifts in baseline pressure.

**Conclusions:**

The baseline pressures of commercial solid ICP sensors can be altered by ESD's at discharge magnitudes that are clinically relevant. Shifts in baseline pressure change the ICP levels visualised to the physician on the monitor screen, and thereby reveal wrong ICP values, which likely represent a severe risk to the patient.

## Background

In patients with brain injury due to traumatic brain injury, stroke, or complications to neurosurgery, the continuous monitoring of intracranial pressure (ICP) is crucial for surveillance [1-3], even though no randomized trials have confirmed the benefit of ICP monitoring in patients with brain injury [4].

Modern ICP monitoring was first introduced by Janny in 1950 [5] and Lundberg in 1960 [6]. While ICP initially was mostly measured from fluid-filled catheters in connection with the ventricular cerebrospinal fluid (CSF), the first ICP micro transducers were introduced in the 1980's [7,8]. The ICP micro transducers most commonly used today include Camino [9] and Codman [10] ICP sensors which were introduced in the 1980's, the Spiegelberg ICP sensor [11] introduced in the 1990's, the Raumedic ICP sensor introduced in the beginning of 2000 [12], and the Pressio ICP sensor [13] introduced more recently. There is an extensive literature on the assessment of these ICP sensors, including bench testing [10,11,13-19] and clinical evaluation [9,12,20-30].

In our hospital, we have particularly addressed the problem of spontaneous shifts in baseline pressure (zero point) that occur during continuous ICP monitoring. Simultaneous monitoring from two ICP sensors placed nearby in the brain demonstrated spontaneous shifts in baseline pressure, which produced differences in ICP even > 20 mmHg [31]. Since the differences in ICP were accompanied by close to identical ICP waveforms, the differences in ICP could be explained by shifts in baseline pressure of technical, not physiological, origin. Similar observations of marked differences in ICP even > 10-20 mmHg despite identical ICP waveform were done when the ICP sensors were placed in different intracranial locations [30,32]. Moreover, during long-term ICP monitoring, sudden shifts in baseline ICP occurred with few hours interval [33]. The reasons for spontaneous shifts in baseline pressure have not been identified.

The present study was undertaken to explore whether or not commercial ICP sensors are affected by electrostatic discharges (ESD's). To our knowledge this topic has previously not been addressed. The issue of electrical safety in hospitals has received much attention since many years [34-37]. The need for increased awareness of electromagnetic interference with medical equipment also was addressed more recently [38]. In the hospital environment, ESD's can be evoked during patient care such as bedding of hospital beds [39]. The frequency and severity of ESD's are affected by numerous factors such as humidity and temperature, the in-house environment (textiles used in clothing, antistatic floor and washing).

In this study, we made an experimental set-up to deliver ESD's to ICP sensors at magnitudes that are clinically relevant. We tested different types of solid ICP sensors that have previously been extensively tested and are still widely used, namely the Codman ICP sensor, and the Raumedic NeuroVent and NeuroDur ICP sensors.

## Methods

### ICP sensors

**Figure 1:**
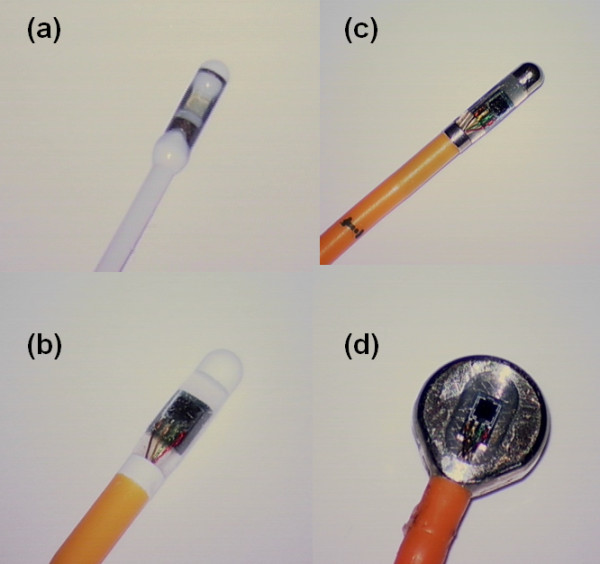
**The different sensors/sensor transducers applied in this study**. In this study we tested the (a) Codman Microsensor, (b) Raumedic NeuroVent P-C, (c) Raumedic NeuroVent P, and (d) Raumedic NeuroDur solid sensors.

The following types of commercially available ICP sensors were tested: Codman ICP MicroSensor (Codman, Johnson & Johnson, Raynham, MA, USA; Figure 1a), Raumedic NeuroVent P-C (Raumedic AG, Münchberg, GE; Figure 1b), Raumedic NeuroVent P (Raumedic AG, Münchberg, GE; Figure 1c), and Raumedic NeuroDur sensor (Raumedic AG, Münchberg, GE; Figure 1d).

Those ICP sensors that had previously been used in patients were stored in closed plastic bags at room temperature.

This study did not include research on humans or animals; ethical approval for the study was not applicable.

### Experimental setup

**Figure 2:**
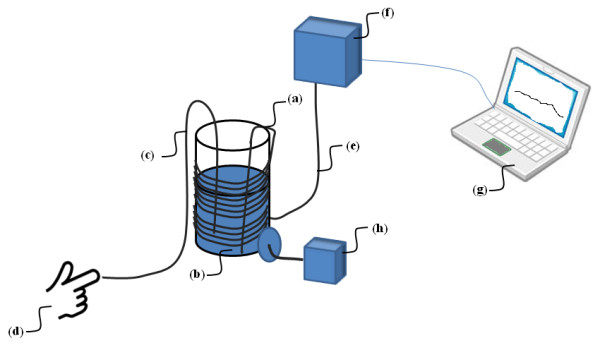
**Experimental set-up**. The ICP sensor (a) was placed in a container (b) containing 0.9% NaCl solution. A metal rod (c) was placed in the 0.9% NaCl solution; thereby the test person (d) could deliver ESD to the sensor (a). The ICP sensor was connected via a cable (e) to the pressure transducer (f), which was the Codman ICP Express for Codman sensors, and Raumedic MPR-1 for the various Raumedic sensors. The pressure transducer (f) was further connected with a laptop computer (g) with Sensometrics software for sampling and storage of the continuous pressure signals. In order to record the ESD delivered to the sensor, a meter (h) was connected with the container to read the magnitude of ESD.

The experimental setup is illustrated in Figure 2. The sensor was placed in a container filled with 0.9% NaCl solution. Also a metal rod was placed in the container, whereby a test-person could deliver ESD's to the sensor. The sensor cable was wrapped around the container in order to compare with the clinical situation, wherein the cable is placed on the patient, resulting in increased capacitance. The wrapping procedure was not done for SensorID's 1-3. The Codman sensor was connected to a Codman ICP Express (Codman, Johnson & Johnson, Raynham, MA, USA), which is a pressure transducer. The Raumedic sensors were connected to a MPR1 Raumedic pressure transducer (Raumedic AG, Münchberg, GE). The continuous ICP signals from the Codman ICP express were sampled at 200 Hz using the Sensometrics pressure logger, which is an analogue to digital converter, and stored on a computer using the Sensometrics^® ^software (dPCom A/S, Oslo). The continuous signals provided by the Raumedic MPR-1 were transferred directly to the computer and stored using Sensometrics^® ^software.

In order to record the electrostatic levels reached, we used the Stat Arc II model 265 (Monroe Electronics Inc., 100 Housel Ave., Lyndonville, N.Y.).

The testing was done in standardized room temperature of 22-23°C.

### Testing of effects of electrostatic discharges

After initiating the pressure recording, the test person was charged using a Metriso 5000 insulation tester (Metrawatt GmbH, Germany) and thereafter touching the metal rod. The test-person was charged in the following sequence: 0.5 kV, 1.0 kV, 2.5 kV, and 5.0 kV. For deliverance of 10 kV, the test person first charged the container to 5kV and then swiftly charged himself to 5 kV opposite polarity before touching the metal rod. The test-person noted whether or not he detected the current impulse being delivered.

The testing of a Codman sensor is shown in Additional file 1, and the testing of a Raumedic sensor is shown in Additional file 2.

### Assessment of leakage current

**Figure 3:**
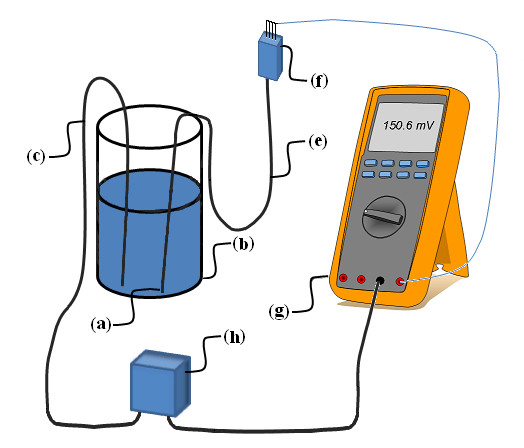
**Set-up for testing of leakage current**. The ICP sensor (a) was placed in a container (b) containing 0.9% NaCl solution. A metal rod (c) was placed in the 0.9% NaCl solution and connected to one side of a 500V source (h). All pins of the ICP sensor connector (f) were connected to the Volts input of the digital multimeter (g) and the multimeter Common input was connected to the other side of the 500V source. The multimeter's input impedance of 10 MΩ thus acted as a shunt resistor providing a current scale of 100pA/mV.

All sensors were tested for leakage current using the Metriso 5000 insulation tester and a Fluke 87 III True RMS Multimeter (in the voltage range). The procedure of testing for leakage current is shown in Figure 3. All leakage current measurements were done at 500V unless otherwise stated; resistance was calculated.

## Results

### ICP sensors

We tested a total of 57 sensors (25 Codman and 32 Raumedic sensors). Six Codman sensors (SensorID's 39 and 49-53) and seven Raumedic sensors (SensorID's 46-48 and 54-57) were new while the other sensors had previously been used in patients. The proportion of ICP sensors with changes in baseline pressure ≥ 2 mmHg is indicated in Table 1.

**Table 1:** ICP sensors tested for altered baseline pressure following ESD

Type of sensor	Number	Number (%) with change baseline pressure ≥ 2 mmHg
Codman ICP Microsensor	25	24 (96)
Raumedic		
NeuroVent P-C	11	11 (100)
NeuroVent P	12	3 (25)
NeuroDur	9	3(33)

**Figure 4:**
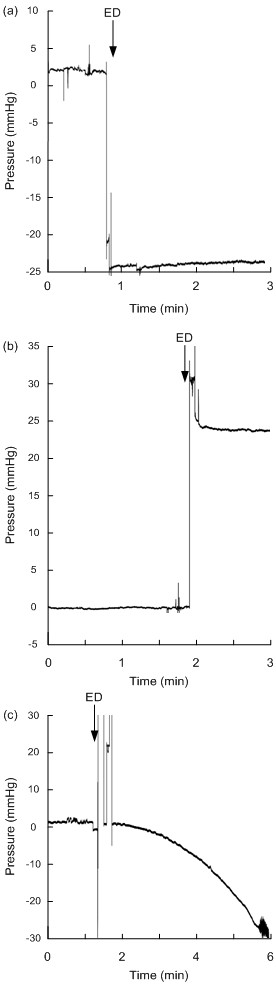
**Continuous pressure signal from Codman sensors before/after electrostatic discharge**. The continuous pressure signals retrieved from Codman Microsensor are shown before and after electrostatic discharges (ESD's) for SensorID 4 (a), SensorID 38 (b), and SensorID 7 (c). Note that sudden changes in baseline pressure occurred for SensorID's 4 and 38, while SensorID 7 showed gradual drift of baseline pressure. The baseline pressure level (mmHg) is indicated on the y axis, and the time line on the × axis levels; the ESD is indicated by an arrow.

The Codman sensors typically responded with sudden shifts in baseline pressure, though gradual drifts in baseline pressure were seen in two sensors (SensorID's 7 and 37). The maximum lasting changes in baseline pressure of Codman sensors are shown in Table 2. Charging the test person to 5 kV caused baseline shift in five sensors (SensorID's 37, 38, 40, 41 and 50), with a measured potential change of 2-5 kV in the 0.9% NaCl solution with the sensor. In the other Codman sensors, changes in baseline pressure occurred when the test person was charged to a 10 kV differential, in which potential changes to the sensor were comparable to that evoked when charging the person to 5 kV. For only one sensor (SensorID 38), we managed to deliver a 10 kV potential change directly to the sensor (taking place after the sensor already had responded to 5 kV). Figure 4 illustrates shifts in baseline pressure in two sensors, and gradual drift of baseline pressure in another. An animation of baseline shift subsequent to ESD's of SensorID 4 is shown in Additional File 3.

**Table 2:** Lasting alterations in baseline pressure of Codman sensors following ESD

ICP Sensor		Baseline pressure (mmHg)		
**Codman**	**SensorID**	**Before**	**After**	**Maximum change^1 ^**

Microsensor	1	-0.7	-13.3	**-12.6**
"	2	0.2	13.4	**13.2**
"	3	-1.5	-9.5	**-8**
"	4	1.7	-24.4	**-26.1**
"	5	-0.02	-1.2	-1.18
"	6	-0.2	-5	**-4.8**
"	7	-0.2	-17.7	**-17.5**
"	8	-0.6	8.7	**9.3**
"	9	11.5	3.8	**-7.7**
"	20	0.5	9.5	**9**
"	36	0	4.8	**4.8**
"	37	8.8	-5.3	**-14.1**
"	38	0	23.2	**23.2**
"	39	-0.7	2.8	**3.5**
"	40	0.4	40	**39.6**
"	41	0	16	**16**
"	42	-6.3	-26	**-19.7**
"	43	1.8	13.1	**11.3**
"	44	-9.3	-0.1	**9.2**
"	45	-4.3	0.02	**4.32**
"	49	-12.2	-23.1	**-10.9**
"	50	-0.1	-15.9	**-15.8**
"	51	-15.3	-0.8	**14.5**
"	52	4.9	12.5	**7.6**
"	53	-0.4	-8.6	**-8.2**

^1^Change in baseline pressure ≥ 2 mmHg (absolute value) is highlighted.

The Raumedic sensors responded differently to ESD's depending on the design of the sensors. While Raumedic NeuroVent P-C was highly unstable to ESD's, the Raumedic NeuroVent P and NeuroDur sensors differed. The maximum lasting changes in baseline pressure for the individual Raumedic sensors are presented in Table 3.

**Table 3:** Lasting alterations in baseline pressure of Raumedic sensors following ESD

ICP Sensor		Baseline pressure (mmHg)		
**Raumedic**	**SensorID**	**Before**	**After**	**Maximum change^1 ^**

NeuroVent P-C	10	18.4	-9.3	**-27.7**
"	11	-0.3	-11.5	**-11.2**
"	12	9.5	3.6	**-5.9**
"	13	0.6	6.9	**6.3**
"	14	-5.4	11.4	**16.8**
"	15	-0.7	-14.1	**-13.4**
"	16	0	18.3	**18.3**
"	18	-1.4	1.1	**2.5**
"	29	-5.7	17.3	**23**
"	46	-12.1	-0.1	**12**
"	57	1.3	-8.3	**-9.6**
NeuroVent P	17	1.6	0.1	-1.5
"	30	-0.2	-0.5	-0.3
"	31	-0.05	-0.9	-0.85
"	32	-0.3	4.7	**5**
"	33	0	9.6	**9.6**
"	34	0.2	0.7	0.5
"	35	0.1	0.5	0.4
"	47	2.5	-0.1	**-2.6**
"	48	0.4	-0.1	-0.5
"	54	0.6	-0.9	-1.5
"	55	0.3	0.5	0.2
"	56	0	-0.1	-0.1
NeuroDur	19	0.1	-0.6	-0.7
"	21	-0.05	-0.1	-0.05
"	22	0	0.1	0.1
"	23	52.4	0.8	**-51.6**
"	24	0.8	0.9	0.1
"	25	0.4	10.7	**10.3**
"	26	0.8	0.3	-0.5
"	27	0.1	0.2	0.1
"	28	0	39	**39**

^1^Change in baseline pressure ≥ 2 mmHg (absolute value) is highlighted.

**Figure 5:**
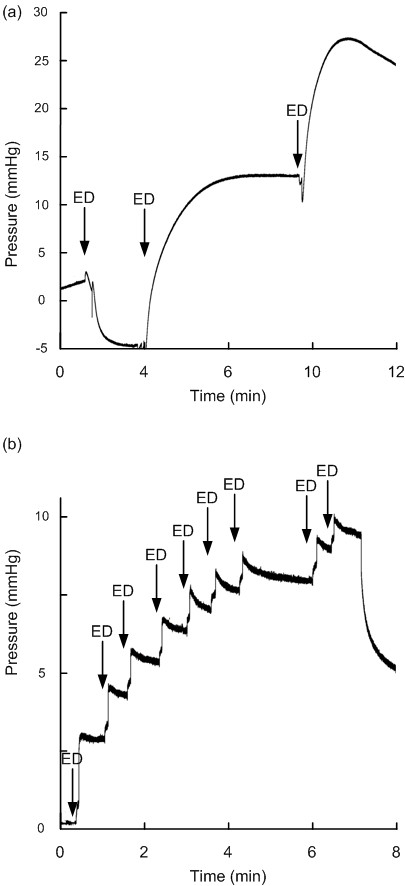
**Continuous pressure signal from Raumedic sensors before/after electrostatic discharge**. The continuous pressure signals retrieved from Raumedic NeuroVent sensors are shown before and after electrostatic discharges (ESD's) for SensorID 14 (NeuroVent P-C; a), and for SensorID 33 (NeuroVent P; b). The baseline pressure level (mmHg) is indicated on the y axis, and the time line on the × axis levels; the ESD is indicated by an arrow. Note that gradual drifts in baseline pressure occurred. For SensorID 33, repeated low ESD's of 0.5 kV (not being sensed by the test person) causes gradual build-up of baseline pressures.

All the Raumedic NeuroVent P-C sensors responded with gradual drifts in their baseline pressure; drifts occurred when the test person was charged to 0.5 kV in 3 sensors (SensorID's 16, 29 and 46), 2.5 kV in 6 sensors (SensorID's 11-15, and 18), and 5 kV in two sensors (SensorID's 10 and 57). The gradual drift of NeuroVent P-C in SensorID 14 is shown in Figure 5a (see also Additional file 4).

The shifts in baseline pressure of NeuroVent P sensors were seen after charging the test person to 0.5 kV in two (SensorID's 33 and 47), and to 2.5 kV in another (SensorID 32). In Figure 5b is illustrated how SensorID 33 gradually changed its baseline pressure following repetitive ESD's of 0.5 kV (an animation of the pressure signal is shown in Additional file 5).

**Figure 6:**
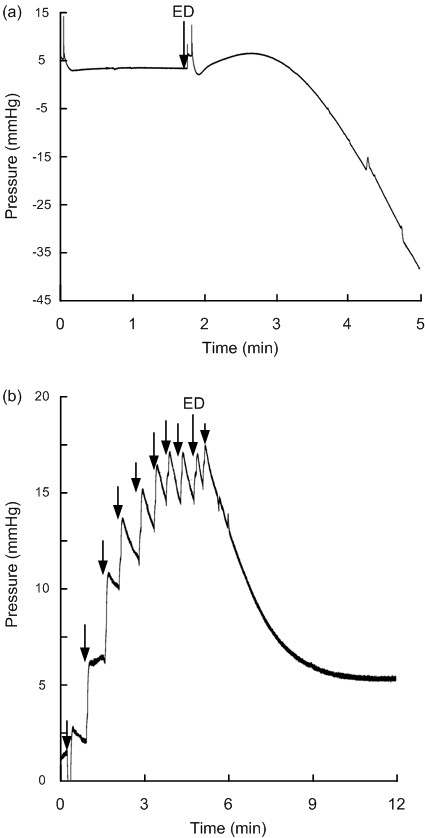
**Continuous pressure signal from Raumedic sensors before/after electrostatic discharge**. The continuous pressure signals retrieved from Raumedic NeuroDur sensors are shown before and after electrostatic discharges (ESD's) of SensorID 28 (a), and for SensorID 25 (b). The baseline pressure level (mmHg) is indicated on the y axis, and the time line on the × axis levels; the ESD is indicated by an arrow. Note that gradual drifts in baseline pressure occurred.

All the three NeuroDur sensors responding to ESD (SensorID's 23, 25 and 28) responded when the test person was charged to 0.5 kV, with a corresponding 0.5 kV potential change in the solution around the sensor. The responses of SensorID's 25 and 28 are shown in Figure 6 (see also Additional file 6).

### Experimental setup

When the test-person was charged to 0.5 kV, the ESD delivered to the sensor was typically 0.5 kV pulse peak. Charging to 5 kV provided for a potential change of 2.5 kV on average (range 2-5 kV). When charging to 10 kV, typically 3.5-4.0 kV change was seen; in a few occasions we observed even 7 kV change (10 kV on one occasion). While ESD's < 3 kV hardly provided any unpleasant sensations, ESD's of about 5 kV gave weak unpleasant sensations, while ESD's ≥ 7 kV provided a sensation which is evident, though not painful, to the test person.

### Leakage current

The results of testing leakage current are presented in Table 4. Leakage current was seen in 1 of the 25 Codman sensors (SensorID 45); this sensor revealed leakage current of up to100 nA with continuous breakthrough.

**Table 4:** Results of testing of current leakage

ICP sensor		Test parameters		
**Type**	**^a^SensorID**	**Test Voltage**	**Leakage Current**	**Resistance**

Codman Microsensor	1	500 V	70 pA	7 TΩ
Codman Microsensor	2	500 V	80 pA	6 TΩ
Codman Microsensor	3	500 V	130 pA	4 TΩ
Codman Microsensor	4	500 V	60 pA	8 TΩ
Codman Microsensor	5	500 V	60 pA	8 TΩ
Codman Microsensor	6	500 V	100 pA	5 TΩ
Codman Microsensor	7	500 V	60 pA	8 TΩ
Codman Microsensor	8	500 V	90 pA	6 TΩ
Codman Microsensor	9	500 V	50 pA	10 TΩ
Raumedic NeuroVent P-C	10	500 V	150 pA	3 TΩ
Raumedic NeuroVent P-C	11	500 V	140 pA	4 TΩ
Raumedic NeuroVent P-C	12	500 V	110 pA	5 TΩ
Raumedic NeuroVent P-C	13	500 V	270 pA	1.9 TΩ
Raumedic NeuroVent P-C	14	500 V	100 pA	5 TΩ
Raumedic NeuroVent P-C	15	500 V	150 pA	3 TΩ
Raumedic NeuroVent P-C	16	500 V	290 pA	1.7 TΩ
Raumedic NeuroVent P	17	143 V	27.6 μA	5.2 MΩ
Raumedic NeuroVent P-C	18	500 V	300 pA	1.5 TΩ
Raumedic NeuroDur	19	142 V	27.6 μA	5.1 MΩ
Codman Microsensor	20	500 V	50 pA	10 TΩ
Raumedic NeuroDur	21	500 V	-	22 Ω
Raumedic NeuroDur	22	142 V	27.6 μA	5.1 MΩ
Raumedic NeuroDur	23	273 V	~16 μA	~17 MΩ
Raumedic NeuroDur	24	142 V	27.6 μA	5.1 MΩ
Raumedic NeuroDur	25	351 V	9.07 μA	38.7 MΩ
Raumedic NeuroDur	26	142 V	27.6 μA	5.1 MΩ
Raumedic NeuroDur	27	142 V	27.6 μA	5.1 MΩ
Raumedic NeuroDur	28	360 V	8 μA	45 MΩ
Raumedic NeuroVent P-C	29	500 V	130 pA	4 TΩ
Raumedic NeuroVent P	30	142 V	27.6 μA	5.1 MΩ
Raumedic NeuroVent P	31	143 V	27.6 μA	5.2 MΩ
Raumedic NeuroVent P	32	335 V	10.5 μA	32 MΩ
Raumedic NeuroVent P	33	500 V	200 pA	2.5 TΩ
Raumedic NeuroVent P	34	273 V	~16 μA	~17 MΩ
Raumedic NeuroVent P	35	500 V	-	100 Ω
Codman Microsensor	36	500 V	80 pA	6 TΩ
Codman Microsensor	37	500 V	70 pA	7 TΩ
Codman Microsensor	38	500 V	70 pA	7 TΩ
Codman Microsensor	**39**	500 V	70 pA	7 TΩ
Codman Microsensor	40	500 V	50 pA	10 TΩ
Codman Microsensor	41	500 V	90 pA	6 TΩ
Codman Microsensor	42	500 V	90 pA	6 TΩ
Codman Microsensor	43	500 V	80 pA	6 TΩ
Codman Microsensor	44	500 V	60 pA	8 TΩ
Codman Microsensor	45	500 V	0.5~100 nA	~
Raumedic NeuroVent P-C	**46**	500 V	210 pA	2.4 TΩ
Raumedic NeuroVent P	**47**	142 V	27.5 μA	5.2 MΩ
Raumedic NeuroVent P	**48**	142 V	27.5 μA	5.2 MΩ
Codman Microsensor	**49**	500 V	100 pA	5 TΩ
Codman Microsensor	**50**	500 V	210 pA	2.4 TΩ
Codman Microsensor	**51**	500 V	130 pA	4 TΩ
Codman Microsensor	**52**	500 V	100 pA	5 TΩ
Codman Microsensor	**53**	500 V	120 pA	4 TΩ
Raumedic NeuroVent P	**54**	143 V	27.6 μA	5.2 MΩ
Raumedic NeuroVent P	**55**	143 V	27.6 μA	5.2 MΩ
Raumedic NeuroVent P	**56**	143 V	27.6 μA	5.2 MΩ
Raumedic NeuroVent P-C	**57**	500 V	320 pA	1.6 TΩ

SensorID^a^: The SensorID's of new and previously non-used sensors are presented with numbers in bold.

The Raumedic sensors differed depending on design. The NeuroVent P-C sensors showed a marginally higher leakage current. Two of the NeuroVent P sensors responding to ESD's (SensorID's 32 and 33) showed leakage current. The three NeuroDur sensors (SensorID's 23, 25 and 28) being affected by ESD's of 0.5 kV, all showed abnormal current leakage.

## Discussion

This study shows that the baseline pressures (zero point) of Codman and Raumedic ICP sensors can be altered by ESD's at magnitudes that are clinically relevant. The observations indicate severe limitations with currently used ICP sensors.

### ICP sensors used for clinical monitoring of ICP

The ICP sensors tested in this study are widely used ICP sensors. Both the Codman [10,14,16,21,22,25,27] and Raumedic [12,19,29,30] sensors have undergone extensive bench and clinical testing. In general, the assessment of ICP sensors has previously focused on long-term-drift of the sensors, sensitivity to temperature changes, and inter-sensor accuracy comparisons [10,11,13-19,22,25].

All the ICP sensors measure pressure relative to atmospheric pressure, which means that they have to be zeroed before measuring ICP. Hence, their zero point equates the atmospheric pressure, and the ICP level displayed on the monitor represents the difference between pressure level within the intracranial compartment and the sensor zero point. It should be noted that in daily clinical practice, various notations are used to refer to the zero point, such as set point, reference pressure, or baseline pressure. In this paper and previous publications [30-33], we have preferred the term baseline pressure, when referring to the zero point of the ICP sensor.

Depending on clinical state, the upper normal threshold of ICP varies between 15 and 25 mmHg [1-4]. Obviously, if the baseline pressure (zero point) spontaneously shifts > 10-20 mmHg, the ICP presented to the physician becomes wrong. Since the continuous monitoring of ICP is done for surveillance of patients with brain injury, e.g. due to traumatic brain injury, stroke complications to brain surgery [1-3], false ICP values represent a likely hazard to the patient. For example, when ICP increases, efforts may be done to reduce the ICP; such efforts include medication, artificial ventilation and surgical procedures.

When the impact of ESD's on ICP sensors previously has not been considered, the reason may be that the issues of non-physiological changes in baseline pressure have not been regarded as a problem in ICP monitoring.

### Electrostatic discharges in the hospital environment

There are different ways to test ESD's; it has also been addressed that there is a need for more standardized methods [40]. The rational for our experimental setup was to best possible test ESD's of clinically relevant magnitudes. Therefore, the ESD's were delivered from a test person, and caused pulse peak discharges to the sensor typically in the range 0.5 - 5 kV. Such ESD's may not be unpleasant to the test person, and are below the levels that can be seen clinically [39,41]. ESD's of magnitudes < 2-3 kV may not even be appreciated by the personnel taking care of the patients. It was previously demonstrated that potentials > 30 kV could be induced on the bed framework when the bedding is pulled from the bed; the degree of charging being dependent on the material of hospital bedding [39]. In comparison, previous tests in our hospital showed that ESD's of 20-40 kV could be seen, depending on the textiles used in clothing (Jensen, Grimnes, unpublished data). Using the test approach described here, we avoided ESD's of magnitudes that are not clinically relevant. Only in a few instances, we managed to deliver 7 kV potential changes to the sensor (10 kV in one sensor that first responded markedly to 5 kV). Accordingly, the voltages referred to here are quite low.

### Different characteristics of Codman and Raumedic ICP sensors

There were some differences between the Codman and Raumedic sensors in their responses to ESD's. The Codman sensors consistently responded to electrostatic changes of 2-3 kV, with sudden shifts in baseline pressure. Gradual drift was only seen in 2 of 25 Codman sensors (8%). These findings compare with our clinical observations of spontaneous alterations in baseline ICP despite unchanged ICP waveform. The observation that baseline pressure was changed maximally > 10 mmHg in 13 of 25 (52%) sensors (and > 20 mmHg in 3 (12%) sensors), indicate that effects of ESD's are of a magnitude that likely would affect patient management.

The Raumedic sensors responded differently depending on their design. Two types of responses were seen, namely gradual drifts and sudden shifts in baseline pressure. While the NeuroVent P-C was completely unstable to ESD's, even at levels of 0.5 kV, the NeuroVent P was less affected. The P-C type incorporates a ceramic coating on the sensor tip while the P type uses titanium. Also the NeuroDur sensor using titanium was more stable, where the tip seemed connected to the sensor with a 5 MΩ resistance. Nevertheless, the observation of alterations in baseline pressure > 10 mmHg in 10 of 32 (31%) Raumedic sensors (> 20 mmHg in 4 of 32 (12.5%) indicate that the effects of ESD's would affect patient management also when using these sensors. In a recent study comparing simultaneous ICP signals from Raumedic NeuroVent and NeuroDur sensors, we encountered average differences between sensors during over-night monitoring > 10 mmHg in 4 of 12 (33%) patients [30].

While leakage current was seen in only one Codman sensor, and no Raumedic NeuroVent P-C sensors, current leakage was seen in 2 of 3 Raumedic NeuroVent P sensors that responded to ESD's, and in all three Raumedic NeuroDur sensors responding to ESD's. The testing of leakage current indicated that in Raumedic titanium sensors (NeuroVent P and NeuroDur) there is an internal 5 MΩ resistance between the metal shell and the connector. Hence, the sensors with resistance different from 5 MΩ (Table 4) might have a broken protection resistor. We found, however, no evidence of sensor damage using microscopy, though damage to ICP sensors may happen both during the implantation and explanation.

### Control of risk associated with ESD's

A major issue with both the Codman and Raumedic ICP sensors is that the health care personnel get no warning about sudden shifts in baseline pressure (zero point) of ICP sensors, or even damage to the ICP sensor during/after implantation. Thereby it is impossible for the physician or nurse to know whether changes in ICP are related to ESD's or not. The Codman sensor cannot be re-zeroed because this is done within the operating room before sensor implantation. The Raumedic sensors, on the other hand, can be re-zeroed after implantation; however, this procedure is not necessarily done by the nurse/physician when ICP is changing.

While the present study focused on effects of ESD's on ICP sensors, the baseline pressure can also be affected by user-related wrong zeroing or even damage to the sensor during implantation, which may not be recognized. Therefore, it can be questioned why modern monitoring systems include no warning. Such warning should be incorporated as part of risk control.

We suggest that a robust way of incorporating risk control is by determining the ICP from the ICP waveform itself. Thereby quality control is accomplished and the issue of baseline pressure alterations is eliminated. The first author previously described a procedure for automatic identification of the cardiac-induced waves in the ICP waveform [42]. Using this approach, the ICP parameters such as the mean ICP wave amplitude (MWA), can be determined from the cardiac induced ICP waves [42]. Since such determination of single wave pressure parameters is done within the ICP signal itself, the analysis results is not affected by changes in baseline pressure. The automatic identification of verified cardiac induced ICP waves also recognizes other ICP sensor-related issues. For example, if an ICP sensor is placed wrong by mistake, artificial waves and no cardiac induced ICP waves will be identified, providing feedback to the user that the ICP signal is erroneous.

## Conclusions

The baseline pressure (zero point) of the Codman and Raumedic ICP sensors can be altered by ESD's at discharge magnitudes that are clinically relevant levels. The shifts in baseline pressure will directly affect the ICP levels visualised to the health care personnel. The alterations in baseline pressure can be extensive (> 10-20 mmHg), thereby revealing wrong ICP values, which subsequently poses a high risk for erroneous treatment.

## Abbreviations

ICP: Intracranial pressure; ESD: Electrical discharge; kV: kilo Volt; CSF: cerebrospinal fluid; MWA: mean ICP wave amplitude; SW: single wave.

## Competing interests

AB reports no conflicts of interest. PKE has financial interest in the software company (dPCom A/S) that manufactures the software (Sensometrics^® ^Research software and Sensometrics^® ^Software), which was used for digital recording of the continuous pressure signals in this study.

## Authors' contributions

Both authors have made substantial contributions to conception and design, acquisition of data, analysis and interpretation of data; and have been involved in drafting the manuscript or revising it critically for important intellectual content. Both authors have read and approved the final manuscript.

## Supplementary Material

Additional file 1 **The testing of a Codman sensor**. The video shows the testing of a Codman sensor. A test person is being charged, and then touches the metal rod, leading the electrostatic discharge to the container filled with 0.9% NaCl Ringer solution, wherein the sensor is placed. A schematic illustration is shown in Figure 2. Click here for file

Additional file 2 **The testing of a Raumedic sensor**. The video shows the testing of a Raumedic sensor. A test person is being charged, and then touches the metal rod, leading the electrostatic discharge to the container filled with 0.9% NaCl Ringer solution, wherein the sensor is placed. A schematic illustration is shown in Figure 2. Click here for file

Additional file 3 **A continuous pressure signal before and after ESD - Codman sensor**. The continuous pressure signal of a Codman sensor (SensorID 4) is shown at a higher velocity (about × 30) than normal. The test person was charged to 10 kV, which delivered an ESD of 5 kV to the sensor. At the time of the ESD, a sudden change in baseline pressure occurs. See also Figure 4a. Click here for file

Additional file 4 **A continuous pressure signal before and after ESD - Raumedic NeuroVent P-C sensor**. The continuous pressure signal of a Raumedic P-C sensor (SensorID 14) is shown at a higher velocity (about × 30) than normal. The test person was charged to 2.5 kV three times, which delivered ESD's of 1-2.5 kV to the sensor. At the time of the first ESD, a slight reduction of baseline pressure occurred, while at the second and third ESD's, the baseline pressures drifted to higher levels. See also Figure 5a. Click here for file

Additional file 5 **A continuous pressure signal before and after ESD - Raumedic NeuroVent P sensor**. The continuous pressure signal of a Raumedic P sensor (SensorID 33) is shown at a higher velocity (about × 30) than normal. The test person was repeatedly charged to 0.5 kV, which delivered ESD's of 0.5 kV to the sensor. At the time of each ESD, a slight increase of baseline pressure occurred. See also Figure 5b. Click here for file

Additional file 6 **A continuous pressure signal before and after ESD - Raumedic NeuroDur sensor**. The continuous pressure signal of a Raumedic NeuroDur sensor (SensorID 28) is shown at a higher velocity (about × 30) than normal. The test person was charged to 0.5 kV, which delivered an ESD of 0.5 kV to the sensor. At the time of ESD, a marked downward drift of baseline pressure occurred. See also Figure 6a. Click here for file
